# Device Functionalities and Technology Acceptance for Innovations in Neonatal Ventilation and Enhanced, Immediate Newborn Care: International, Multicenter, Web-Based Survey Study

**DOI:** 10.2196/64701

**Published:** 2025-05-28

**Authors:** Anna-Sophie Käferböck, Meggy Hayotte, Daniel Sieber, Martin Pillei, Martin Wald

**Affiliations:** 1 Medical Sciences, Paracelsus Medical University, Strubergasse 21, Salzburg, 5020, Austria, 43 5122070 ext 4444; 2 LAMHESS, Université Côte d’Azur, Nice, France; 3 Department Medical and Health Technologies, MCI | The Entrepreneurial University, Innsbruck, Austria; 4 Industrial Engineering and Management, MCI | The Entrepreneurial University, Innsbruck, Austria; 5 Division of Neonatology, Department of Pediatrics and Adolescent Medicine, University Hospital Salzburg, Paracelsus Medical University, Salzburg, Austria

**Keywords:** infant, neonatology, medical device, respiration disorders, pulmonary ventilation, medical engineering, technology acceptance model, technology acceptability, resuscitation, critical care

## Abstract

**Background:**

A substantial number of newborns face postdelivery respiratory issues annually. Current ventilation devices in immediate newborn care lack integrated sensors and supporting mechanisms for medical professionals. This is a potential field of improvement, as safe ventilation relies on accurate pressure administration in current t-piece resuscitators. As the needed support during the process is currently limited, it highlights the demand for innovations in neonatal ventilation technology to improve efficacy and reduce potential errors.

**Objective:**

The objective of the study was to facilitate collaboration between medical and engineering experts to evaluate the critical factors for the successful implementation of an innovative ventilation technology in clinical immediate newborn care. Incorporating the views of medical professionals into the survey is expected to offer valuable insights to engineers for subsequent technological refinement.

**Methods:**

An international multicenter online survey was conducted among 51 neonatal health care professionals in the DACH region (Germany, Austria, and Switzerland) in order to (1) assess the specific functionalities required in a neonatal ventilation assistant in immediate newborn care from a medical technology viewpoint, (2) characterize the acceptance of such a device as support tool using the extended technology acceptance model, and (3) identify further steps toward integration of such technologies.

**Results:**

According to the results, a visual representation of the current mask leakage and tidal volume is an essential feature. Integrating alarms in visual rather than audible form when limit values are exceeded is preferable. In contrast, medical professionals ranked an external control using a foot pedal as the least necessary feature. Based on the findings, acceptance constructs of the neonatal ventilation technology were moderately scored. Perceived usefulness (β=.76, *P*<.001) was the main predictor of the behavioral intention to use such a supportive instrument.

**Conclusions:**

There is an evident willingness to integrate sophisticated support techniques into a neonatal ventilation device for immediate newborn care.

## Introduction

The probability of survival of newborns depends on the extent to which they can adapt to the new environment and living conditions after delivery. Nevertheless, 3% of premature or newborn infants required respiratory support in the form of mask ventilation in the first few minutes of life [1], with approximately 1% of infants requiring postnatal resuscitation [2]. The lungs and associated tissue need to develop in the first few minutes of life before they are fully functional [3]. This period is highly critical, as excessive positive pressure ventilation can lead to damage to the lung tissue [3].

This issue is further compounded by the stress [4] experienced by medical personnel during a resuscitation scenario or ventilation procedure, which disrupts their typical level of physical performance and further exacerbates the situation. This can lead to inaccurate assessment of color [5] and heart rate [6]. One study [7] showed that despite a constant level of peak inspiratory pressure (PIP), maximum pressure on the lungs during inhalation, there was a range in the tidal volume administered, with the rationale that chest movement may not be accurately detected [8]. In such instances, the utilization of medical devices becomes paramount, as they have the potential to alleviate the psychological burden on health care providers.

The choice of ventilator used by medical personnel makes a difference in the actual quality of neonatal ventilation [9,10]. One study [11] has determined that the currently used technology (t-piece) may result in excessive ventilation pressures for newborns. Encounter emerging criticism regarding the inaccurate administration of ventilation pressures through t-piece devices [12], it is fundamental to design upgraded products in medical care to enhance safety in newborn life support.

This study aimed to assess essential device features for neonatal ventilation during immediate newborn care, as existing research addresses this aspect only in a limited way. The goal is not to replace the clinician but to enhance support through predefined functions that minimize potential errors and improve patient outcomes. The primary objective was to identify neonatologists’ acceptance and need for technology that aids neonatal ventilation in the delivery room. The study sought to establish a collaborative interface between engineers and neonatologists, thereby aligning their perspectives and guiding the development of technology in this domain. A central objective was to address potential misunderstandings between medicine and technology proactively.

## Methods

### Recruitment

This survey was conducted online using the platform LimeSurvey (LimeSurvey Cloud Version 6.3.0, LimeSurvey GmbH). The first page of the survey stated the objective; the inclusion criteria, which were limited to individuals with (1) completed medical degrees and (2) a valid license to practice in neonatology; and the imperative need for their participation. The second page was dedicated to the information leaflet and the electronic consent form. Sociodemographic data and experiences with neonatal ventilation (ie, inquired about how participants would assess their personal experience in neonatal ventilation, the frequency of their exposure to this practice, and their familiarity with specific ventilation equipment used in the delivery room) were then asked, followed by the evaluation of particular requirements and assessment of the acceptance of a possible prototype of a medical device in the area of newborn ventilation in immediate newborn care.

Individual responder contacts were not available, therefore, cluster sampling was used to target the responders by their neonatology departments. The survey link was sent to a total of 351 neonatology departments in the DACH region (Germany, Austria, Switzerland), which provided contact information to the Department of Neonatology of the University Hospital Salzburg. Neonatology departments were asked to distribute the link to the survey to their medical experts using a prepared email. Departments were contacted 3 times to improve the response rate. Respondents were given the option to answer the questionnaire in either German or English. Data collection took place in September and October 2023.

### Measurement

The survey consisted of two parts: (1) evaluation of specific requirements, suggested by a consensus of experts in neonatology of the University Hospital Salzburg, for a medical device in the area of newborn ventilation in immediate newborn care; and (2) evaluation of the acceptance of a possible prototype of this technology based on the extended technology acceptance model (TAM) [13].

To assess the acceptance of the ventilation device for premature and newborn patients, we used the TAM constructs [13], extended with the constructs of trust and risk [14], and the construct of social influence [15], which were adapted for this specific technology. In this adaptation, we substituted “information technology” [16] with “Neonatal Ventilation Device in Immediate Newborn Care.” Data were gathered through an adapted TAM questionnaire containing 29 items distributed across 6 dimensions—perceived usefulness (6 items) [17], perceived ease of use (6 items) [18], behavioral intention to use (4 items) [16], social influence (5 items) [17], trust (3 items) [14], and perceived risk (5 items) [16]. The German version was translated based on existing versions [19] and adapted for the technology studies.

A pretesting phase preceding the study was conducted to ensure the comprehensibility of the included questionnaire items. Participants rated questions on a scale of 1 to 7, where “1” represented “not understandable” and “7” denoted “completely understandable.” All items were above a cutoff of 4 at the end of the iterative phase, with an average rating of 5.4/7 (SD 1.3), indicating a good understanding of the individual items when applied to the response scale. Respondents (n=19), both academic and medical professionals, carried out this assessment.

The survey used a 7-point Likert scale for evaluation, where “1” indicated “strongly disagree” and “7” represented “strongly agree.” The original survey version is attached in the Multimedia Appendix 1. In our sample, TAM dimensions showed high internal consistency (Cronbach α ranged from 0.73 to 0.94) [20]. Overall, the survey was structured according to that suggested by Burns et al [21] in their work with clinicians, with a cluster sampling design, a mixture of free-text and structured questions (mainly based on validated questionnaires), as well as a pretesting phase to ensure comprehensibility of the survey.

### Statistical Analysis

The statistical analysis was undertaken using Excel (Microsoft Excel 2016, Microsoft Corp) and SPSS (IBM SPSS Statistics 29.0.0, IBM Corp). Means, medians, and SDs were used to present continuous measurements, and results from categorical measurements are shown as numbers and proportions. Multivariate ANOVAs were performed to examine differences in the perceptions of the technology requirements as a function of the participants’ characteristics (gender, age, and experience). Furthermore, 2 authors (ASK and DS) were involved in the analysis of the qualitative data by deductive analysis using the software MAXGDA (MAXGDA 24, VERBI – Software. Consult. Sozialforschung. GmbH).

### Ethical Considerations

The study was conducted in accordance with local ethical guidelines as stipulated by the seventh revision of the Declaration of Helsinki. Ethical clearance was obtained from the Ethics Committee Salzburg (415-EALL/4/207/6‐2024). Informed consent was obtained from all participants at the start of the study, and their data were analyzed anonymously. No forms of financial compensation were provided to the participants in this study.

## Results

### Sample Characteristics

A total count of 131 clicks was captured, 82 were identified as eligible attempts to respond by providing consent to participate, resulting in 51 valid responses. The survey was undertaken in German only. Physicians of pediatrics and adolescent medicine specializing in neonatology contributed to all responses. No anesthesiologists engaged in the field of neonatology participated, despite meeting the inclusion criteria. Participants reported their personal experience with neonatal ventilation with a mean of 6.0/7 (SD 1.2, median 6.0, IQR 5.00-7.00) and indicated the frequency of exposure with a mean of 5.8/7 (SD 1.4, median 6.0, IQR 5.00-7.00). Refer to Table 1 for detailed participant characteristics.

**Table 1.** Sample characteristics (n=51).

Variable and level		Count and proportion, n (%)	
Sex			
	Male	27 (52)	
	Female	24 (47)	
Age (years)			
	18‐29	1 (1)	
	30-39	9 (17)	
	40-49	19 (37)	
	50-59	16 (31)	
	60-69	6 (11)	
	70-79	0 (0)	
Educational level			
	Ongoing residency	8 (15)	
	Completed residency	3 (5)	
	CR^a^ with ongoing specialization	4 (7)	
	CR with completed specialization	35 (68)	
Employment status			
	Full-time employment	38 (74)	
	Part-time employment	12 (23)	
	Retired	1 (1)	
Ventilation experience (1=basic knowledge, 7=high expertise)			
	1	0 (0)	
	2	0 (0)	
	3	2 (3)	
	4	5 (9)	
	5	8 (15)	
	6	13 (25)	
	7	23 (45)	
Ventilation frequency (1=never, 7=very often)			
	1	0 (0)	
	2	1 (1)	
	3	3 (5)	
	4	6 (11)	
	5	9 (17)	
	6	8 (15)	
	7	24 (47)	

a CR: completed residency in pediatrics and adolescent medicine.

The ventilator from Paykel Healthcare, which operates under the name Perivent in German-speaking countries, but is also called Neopuff, was named the most frequently used device (n=44). The participants had the opportunity to select multiple devices simultaneously, depending on which ones they were currently using and with which they had previous experience. Furthermore, 2 of the devices mentioned were originally not designed by the manufacturers for ventilation of newborns in the delivery room, but for continuous ventilation in an intensive care setting. One of the devices cited is not intended for use in the immediate newborn care setting by the manufacturer, as it is specifically designed as a monitoring system for the neonatal ventilation process. Further details on the devices used for ventilation in the delivery room setting are illustrated in Table 2.

**Table 2.** Currently used ventilation devices in immediate newborn care.

Device designation	Manufacturer	Country	Count, n
Perivent or Neopuff	Fisher & Paykel Healthcare	New Zealand	44
Giraffe Stand-alone Infant Resuscitation System	GE HealthCare	Canada	15
Giraffe Warmer	GE HealthCare	Canada	14
Neo-Tee	MedCare Visions GmbH	Germany	14
Resuscitaire	Drägerwerk AG & Co. KGaA	Germany	13
rPAP	Inspiration Healthcare	England	8
Stephan F120	Fritz Stephan GmbH	Germany	4
Panda Warmer ResusView	GE HealthCare	Canada	4
Babyroo TN300	Drägerwerk AG & Co. KGaA	Germany	3
Neo100^a^	Monivent AB	Sweden	3
Leonie Plus^b^	Löwenstein Medical SE & Co. KG	Switzerland	3
Hamilton-C1^b^	Hamilton Medical AG	Switzerland	2
Resusci Flow with Blender Unit 104 E Type	Atom Medical Corp	Japan	1
PNEUPAC BabyPAC100	Smiths Medical, Inc	United States	1

a Monitoring device for ventilation in immediate newborn care.

b Not designed for neonatal ventilation in immediate newborn care.

### Device Functionalities

**Figure 1.**
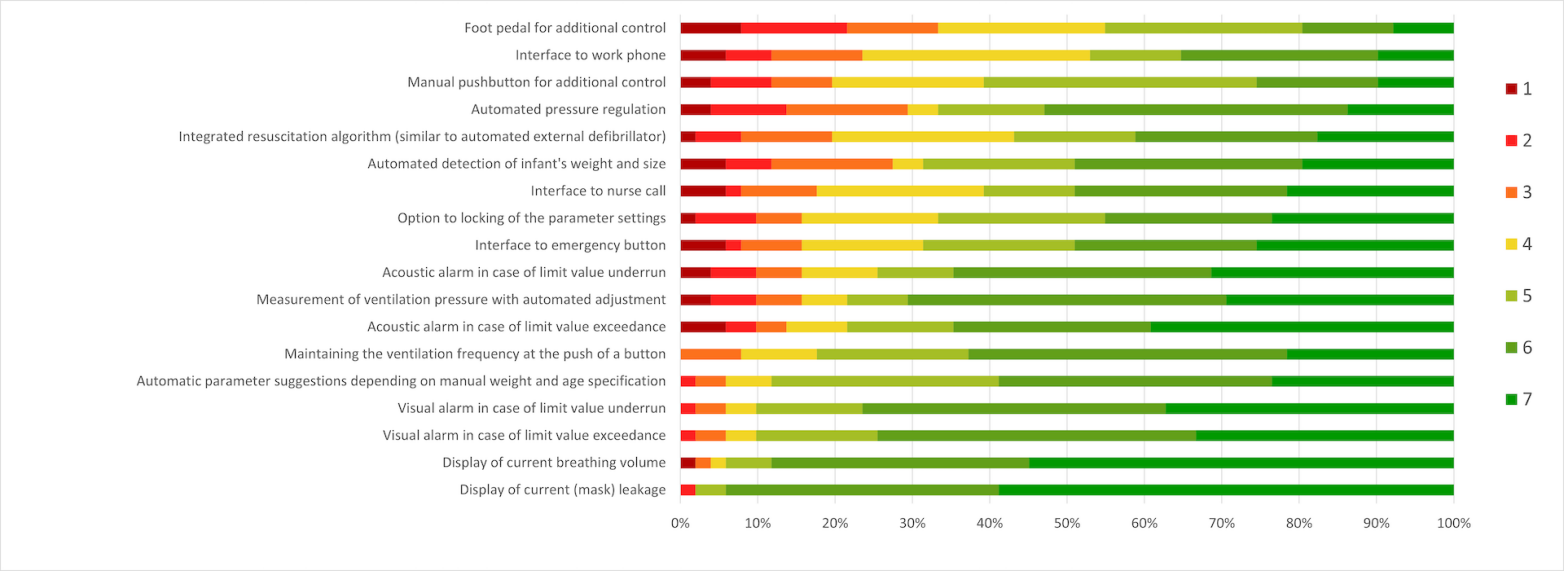
Assessment of requirements for a ventilation device in immediate newborn care (n=51; no significant gender [*F*_18,32_=1.40; *P*=.20], age [*F*_72,128_=0.95; *P*=.60], or experience [*F*_72,128_=1.01; *P*=.47] differences were shown based on multivariate ANOVAs).

The participants rated the listed device features in terms of their future needs for implementation. Figure 1 illustrates the percentages of each rating score in a bar chart for each requirement. In addition to these predefined ratings, the participants were asked to what extent automation of workflows of neonatal ventilation at the push of a button might make sense: “1” represented a manual system, where the whole responsibility lies with the individual, whereas “7” indicated a fully automated system where the person is solely responsible for monitoring the device. The results show an average score of 4.2/7 (SD 1.7, median 4.0, IQR 3.00-5.00).

In addition to the predefined device requirements that participants were asked to rate, respondents were also provided with the opportunity to express their opinion on various requirements through a free-text field (n=18). Accordingly, a neonatal ventilation device used in the delivery room should include a “display of fraction of inspired oxygen” (n=3), along with the output of the “measurement of End-tidal carbon dioxide” (n=2) and the display of a “pressure-volume curve” (n=1). Another potential integration into such technology would be the capability for “humidification” (n=2) and “warming” (n=1) of breathing gases. Furthermore, features such as “time measurement” (n=1), “tidal volume per body weight” (n=1), and “alarms in the event of technical defects” (n=1) should be incorporated into this device. Another suggested feature could be an “automated start procedure with first ventilation” (n=1). A “memory function” (n=1) of the entire process would be beneficial for postintervention analyses. Finally, some participants expressed the preference “not to rely exclusively on the device with integrated technology” (n=2), emphasized the importance of considering “users’ experience” (n=1), and suggested that, in case of uncertainty, preset parameters can be “manually overwritten” (n=4).

### Technology Acceptability

The participants expressed moderate levels of acceptance for the ventilation device in immediate newborn care based on the extended TAM (mean perceived usefulness 5.2, SD 1.1; mean perceived ease of use 4.8, SD 0.7; mean social influence 4.6, SD 1.1; mean trust 4.3, SD 1.2; mean risk 3.0, SD 0.9; mean behavioral intention 5.1, SD 1.2). Perceived usefulness, perceived ease of use, social influence, trust, and behavioral intention were all rated positively, while risk was rated as negligible.

### Difficulties in Usage

#### Overview

In addition to the possible functions of such a device in the delivery room, participants were free to comment on any difficulties that might arise when using it. The ratings were divided into difficulties with first use (n=47), repeated use (n=25), and routine use (n=31).

#### First Use

Possible difficulties when using this type of technology for the first time can be divided into 6 categories based on the responses. Potential problems could lead to “incorrect operation” (n=10) during the process due to “personal uncertainties” (n=13) with the new technology. In addition, the “complexity of the system would require training” (n=9). There is also a risk of “relying on device support” (n=3) without additional follow-up checks of essential parameters. In addition, there is still the possibility of “technical malfunctions” (n=1) when using the system for the first time. In the “other” category (n=6), the following comments were made: on the one hand, that “curiosity to try something new” (n=1) arises personally when using the device for the first time, which allows “sensible setting of parameters” (n=1) and is “easy to use” (n=2). On the other hand, participants could imagine that the “individual assessment of the newborn” (n=1) plays a role in first-time use and that “focusing on the technology could lead to a reduction in observation of the child” (n=1).

#### Repeated Use

With repeated use of a new type of neonatal ventilation technology in the delivery room setting, the participants mentioned the difficulty that “clinical knowledge could be lost” (n=7) if a particular “reliance on the device” (n=3) was established. What would exist would be an “incorrect use” (n=2) with an “increased training effort” (n=4) in order to achieve a specific “safety in use” (n=4). However, after repeated use, the participants could already imagine having “fewer problems with increasing routine” (n=3). The “robustness of the device” (n=1) should, therefore, not be underestimated, as should “overly liberal use” (n=1) during this process.

#### Routine Use

In the course of routine use of such an assistance tool, the participants stated, among other things, the difficulty that users could “lose their clinical judgment” (n=11) due to increasing “familiarization with the technology” (n=5). However, “easy handling” (n=3) with little to “no” (n=5) issues in handling should arise in the course of routine. This technology could provide more “time for decisions relevant to care” (n=1), but there would still be the option of “no significant improvements” (n=1) in the overall process, for example, by “ignoring alarms” (n=2) because personal “expectations” (n=1) are different. In addition, “additional costs” (n=2) could be incurred for materials or upgrading an existing device.

#### Life-Threatening Malfunctions

In addition to the information on possible issues and difficulties in using such a respiratory assistant, the participants could also give their thoughts on life-threatening malfunctions (n=39). Comments were primarily related to “malfunctions in the area of ventilation pressure” (n=15), with incorrect outputs, both too high and too low, in the area of PIP and positive end-expiratory pressure (PEEP), as well as possible difficulties adjusting to the patient’s conditions. A “total failure” (n=9) of the entire system for no foreseeable reason and without a workaround (eg, switching to manual mode) was the second most common unacceptable malfunction. “Personal confusion” (n=8) when using such a tool can also lead to life-threatening situations, which must be considered during development. Incorrect “measurements of the system” (n=5), starting with incorrect detection of the child’s weight and the associated miscalculation of ventilation pressures, must be prevented at all costs. In addition, a “too long activation time” (n=1) before ventilation can take place, and “complicated handling” (n=1) can pose a life-threatening risk.

### Additional Comments

In the final part of the questionnaire, there was also the option to provide general comments on medical technology in neonatal ventilation that the participants wished to contribute in the course of the survey. A total of 29 comments were received, with one response stating that “no” further issues were brought forward. In this section, the participants were concerned that “manual mode” (n=8) could be used to override any automated default settings or, if in doubt, switch them off. Such a system should be “easy to use” (n=6) in order to support both newcomers to the profession and neonatologists with many years of experience in their work. In addition, users should receive good and regular “training” (n=4) on the assistance tool in order to ensure that the familiarization phase and the assessment of therapy needs are of high quality. In the area of technical functionalities, functions such as “warming and humidification” (n=3) of the respiratory gas, “coupling with vital parameters” (n=1) and a “display of the ventilation strokes applied” (n=1) as well as a “sufficient battery” (n=1) and “alarm management” (n=1) should be considered. Depending on the different hospitals and clinics, there is also a difference as to which “group of people would use such technology” (n=1). The option to switch to a “mobile and portable version” (n=1) would avoid possible structural difficulties.

## Discussion

### Principal Results

This paper describes the results of a questionnaire survey in the field of ventilation technology for neonates in immediate newborn care. The responses highlighted the need for support mechanisms during the neonatal ventilation process, as participants rated the desired level of automation of such a technology as 4.1 out of 7, representing a medium to high degree of automation. The guidelines for neonatal care [22] further indicate that while situations requiring neonatal resuscitation are rare, the routine expertise to provide optimal quality is often lacking. Our findings should therefore serve as a starting point to support neonatologists in their practice in the best possible way, in terms of making high efforts in development in pressure administration, intuitive device design for easy handling, and display of critical vital parameters.

The current state-of-the-art neonatal ventilation in the delivery room uses a tube system containing a t-piece with a mask or tube plugged in. This system connects to an air-powered ventilator, where a specific oxygen saturation is set. A valve on the t-piece allows control of PEEP to manage lung pressure at the end of expiration. However, it lacks integrated monitoring of parameters such as tidal volume, ventilation pressure, in particular the PIP, and the determination of pressure loss due to mask leakage.

Our results are consistent and confirm a previous study [23] examining the influence of human factors during this process and improving the situation with assistive tools, such as integrating a display of current (mask) leakage, rated as the leading requirement. Free-text comments from individuals in our research support this trend. On the other hand, it is essential to highlight that one particular study [24] demonstrated that individuals with greater expertise in this domain exhibit an elevated mental workload and encounter higher cognitive demands throughout the process compared with inexperienced individuals using an assistive tool. In order to enhance personal performance during neonatal ventilation or resuscitation, the equipment to be used should be kept to a minimum in order to avoid fixation errors [25].

A topic addressed in the free-text comments pertained to the potential for “manual overwriting” of the device and the “incorrect usage during initial operation.” According to the established standards, specifically the norm titled “Medical Devices -- Part 1: Application of Usability Engineering to Medical Devices” (IEC 62366-1:2015), such concerns should be anticipated during the manufacturing process and are fundamental prerequisites for any medical device. The operation of a medical device is intended to be seamless for the user and should minimize the awareness of the underlying technical interactions. It is recommended to carry out so-called usability tests to obtain such device properties. These tests are based on a specific scheme which, in addition to other considerations, should bring to light the problems mentioned earlier and provide opportunities to improve them [26].

It is important to note that this study focused on neonatologists, as they are the specialists in the ventilation of newborns. However, it is also worth considering that other medical staff, such as midwives, nurses, and anesthesiologists, could use the designed device. It is equally essential to acknowledge that some of the aforementioned devices for neonatal ventilation are not intended for immediate newborn care but rather for continuous ventilation in an intensive care setting or monitoring purposes only. This reinforces the importance of this work in providing an in-depth understanding of the needs of physicians for the development of assistive technologies specifically dedicated to neonatal ventilation in immediate newborn care.

### Limitations

This work has some limitations, one of which is that the link to the survey was solely shared with neonatology facilities in German-speaking countries (DACH region). Therefore, only German-speaking neonatologists participated in the survey. No physicians with a background in anesthesia specialized in neonatology took part in this study. It could be speculated that they do not feel enough concerned by the survey. Second, to ensure a clear understanding of the questionnaire study, a pretesting phase was carried out on a small scale to assess the comprehensibility of the questions within the target group. Although considerable efforts were made to obtain a sufficient number of replies by broadly diffusing the survey, it ultimately resulted in a limited number of valid responses. It may be that not every center contacted and disseminated the survey due to the high volume of inquiries that doctors receive. It is advisable to consider this potential limitation when interpreting the results.

Third, there was a huge difference in group sizes of the sample in relation to the educational level, where the group of fully qualified physicians of pediatrics and adolescent medicine with completed specialization in neonatology were overrepresented. Fourth, the method of free text response used to examine complementary user requirements could have been better examined through qualitative interviews. Based on these findings, future research could include qualitative interviews for a first prototype.

### Comparison With Previous Work

Simulation training using a respiratory function monitor is already being conducted to further assist health care professionals by providing real-time information on the ventilation process [27]. However, certain studies [28-30] indicate a tendency for individuals to shift their focus from the infant to the monitor. This underlines the importance of a broad analysis of technologies in the delivery room. Our findings are in line with the idea that additional information, such as the display of current mask leakage and tidal volume, during the neonatal ventilation process may be beneficial, as well as with the experience of resuscitators using a respiratory function monitor [23]. However, additional information should not distract from the child nor overwhelm the caregiver. Considerations for individual functions should prioritize visual alarm functions over auditory ones to minimize potential distractions as well as to regain focus on the child.

### Conclusions

The integration of supportive mechanisms into neonatal ventilation within the context of immediate newborn care is essential for the assistance of medical personnel. The acceptance of neonatologists of a device with additional functionalities and integrated sensors indicates a promising outlook. The objectives are clearly defined—to enhance the safety, efficacy, and quality of neonatal ventilation by minimizing human error and optimizing the overall care provided. The priorities encompass the improvement of device functionality, the assurance of user-friendliness, and the reduction of ventilatory complications. The analysis of technology acceptance reveals significant potential for future incorporation into existing systems.

The participation of physicians in the development process is of utmost importance. Their clinical expertise guarantees that the technology is aligned with real-world needs, remains intuitive, and integrates seamlessly into established protocols. Engaging with clinicians from the initial stages establishes a connection between medical and technological necessities, thereby ensuring that the technology improves patient care and aids clinicians in the delivery room.

## Supplementary material

10.2196/64701 Multimedia Appendix 1 Detailed questionnaire for medical personnel in neonatology.

## Abbreviations

DACH region: Germany, Austria, and Switzerland
PEEP: positive end expiratory pressure
PIP: peak inspiratory pressure
TAM: technology acceptance model
