# Supplementary material for: Device Functionalities and Technology Acceptance for Innovations in Neonatal Ventilation and Enhanced, Immediate Newborn Care: International, Multicenter, Web-Based Survey Study
Source: JMIR Hum Factors. 2025 May 28;12:e64701. doi: 10.2196/64701 (PMC12136511; doi:10.2196/64701)
Supplement: Multimedia Appendix 1 [file humanfactors-v12-e64701-s001.pdf]

neonatal ventilation in primary care

The main objective of this study is to survey healthcare professionals in the field of neonatal medicine and examine their opinions regarding the research project.
There are 18 questions in this survey.

Informed Consent for Study Participation

1) Title of the Study

Assessment of the demand for a medical device to support neonatal ventilation in initial care

2) Opening Statement

We hereby cordially invite you to participate in the research project in the area of neonatal ventilation. Before you make a decision regarding your participation, it is essential that you are fully informed about the purpose and content of the present study. We therefore ask you to read the following information carefully before indicating your willingness to participate.

3) Why is this study being conducted?

This study is part of an interdisciplinary research project between MCI | Die Unternehmerische Hochschule® (MCI) & Gemeinnützige Salzburger Landeskliniken Betriebsgesellschaft mbH (SALK).

4) Why have you been selected?

The decision to select you as a participant for this study is based on your compliance with the criteria of the target group, which is defined as follows:

professional activity in neonatology with successfully completed medical studies

5) What do you need to do if you decide to participate?

If you decide to participate in this study, you will be asked to complete a questionnaire. The purpose of this questionnaire is to collect sociodemographic data and to investigate the acceptability of a medical device to support neonatal ventilation in primary care. In addition, the functional requirements and risks in the field of ventilation technology will also be investigated.

6) How much time will it take to attend?

Filling out the questionnaire will take approximately 10-15 minutes of your time.

7) Is participation mandatory?

Participation in this study is solely on a voluntary basis. If you do not wish to participate in this study, you can opt out at any time.

8) What are the advantages and / or disadvantages of participation?

To our knowledge, there are no known risks associated with this study. However, if you feel uncomfortable with some questions, the supervisor of the study, Anna-Sophie Käferböck, BSc MSc, is available for you to contact: *anna-sophie.kaeferboeck@mci.edu*

9) Will your participation in the study remain confidential?

With your consent to participate in this study, all responses you provide will be treated anonymously. The information you provide as part of your participation will not be shared with third parties.

10) What is the further procedure?

To ensure that you understand the expectations placed on you and are aware of your rights in this process, we ask that you accept the consent form below before proceeding.

If you express interest in participating, we now ask you to complete the questionnaire below.

I hereby confirm that it has been explained to me in detail that my participation in this study will involve completing a questionnaire and will take approximately 10 minutes of my time.

I agree that the data collected will be treated anonymously and will only be made available to the research team for study purposes.

I agree that although the data recorded will be treated anonymously, it will not be shared with anyone outside of said research team.

I agree that participation in this study is entirely voluntary and that I may withdraw my participation at any time without giving any reason. In the event that I withdraw my participation, all data that resulted from my participation will be removed unless it has already been made public.

I have been informed that I can ask questions at any time (*anna-sophie.kaeferboeck@mci.edu*). I have the right to withdraw my participation at any time and to discuss possible concerns with the supervisor of the study.

I consent to my data being used for scientific purposes and agree to the publication of my data in anonymized form in scientific publications.

I have been informed that I may contact the research supervisor after completion of the study to obtain copies of all publications related to the current study.

I acknowledge that all information provided by me as part of my participation will be collected anonymously and therefore cannot be traced back to me.

I hereby consent to participate in this study, which will be conducted by Anna-Sophie Käferböck, BSc MSc (anna-sophie.kaeferboeck@mci.edu) and supervised by *Assoc. Prof. Dr. med. univ. Martin Wald, Asst. Prof. Dr. Dipl.-Ing. Daniel Sieber* & *FH-Prof. Dr.-Ing. Martin Pillei, BSc MSc*.

*

Please choose **only one** of the following:

◯ Yes

◯ No

Sociodemographics

The collection of personal information is critical to provide a valid and meaningful data set. This information allows us to capture demographic characteristics of participants such as age, gender, occupation, and experience in neonatal care. Such characteristics can potentially influence perceptions of the device and help us identify patterns and trends in responses.

What is your age? *

Choose one of the following answers
Please choose **only one** of the following:

- ◯ 18-29 y.
- ◯ 30-39 y.
- ◯ 40-49 y.
- ◯ 50-59 y.
- ◯ 60-69 y.
- ◯ 70-79 y.

To which gender do you assign yourself? *

Choose one of the following answers
Please choose **only one** of the following:

- ◯ male
- ◯ female
- ◯ diverse

What is your educational status?

*

Choose one of the following answers
Please choose **only one** of the following:

- ◯ ongoing residency in pediatrics & adolescent medicine
- ◯ ongoing residency in anesthesiology
- ◯ completed residency in pediatrics & adolescent medicine
- ◯ completed residency in anesthesiology
- ◯ completed residency in pediatrics & adolescent medicine with ongoing specialization in neonatology
- ◯ completed residency in pediatrics & adolescent medicine with completed specialization in neonatology

What is your current employment status?

*

Choose one of the following answers
Please choose **only one** of the following:

- ◯ full-time employment
- ◯ part-time employment
- ◯ jobseeking
- ◯ retired
- ◯ studying
- ◯ unable to work

Which clinic do you work in? *

Only answer this question if the following conditions are met:
((WorkStatus.NAOK == "Full" or WorkStatus.NAOK == "Half"))

Please write your answer here:

How would you rate your knowledge and experience of neonatal ventilation?

*

Choose one of the following answers

Please choose **only one** of the following:

- 1 - Basic Knowledge
- 2
- 3
- 4
- 5
- 6
- 7 - High Expertise

How often are you in touch with neonatal ventilation in initial care?

*

Choose one of the following answers

Please choose **only one** of the following:

- ◯ 1 - never
- ◯ 2
- ◯ 3
- ◯ 4
- ◯ 5
- ◯ 6
- ◯ 7 - very often

Which device do you currently work with or do you have experience with?

*

Select all that apply

Please choose **all** that apply:

- ☐ Giraffe Stand-alone Infant Resuscitation System, GE HealthCare, Canada
- ☐ Giraffe Warmer, GE HealthCare, Canada
- ☐ Perivent™ / Neopuff™, Fisher & Paykel Healthcare, New Zealand
- ☐ Babyroo® TN300, Drägerwerk AG & Co. KGaA, Germany
- ☐ Resuscitaire®, Drägerwerk AG & Co. KGaA, Germany
- ☐ rPAP™, Inspiration Healthcare, United Kingdom
- ☐ Resusci Flow with Blender Unit 104 E Type, Atom Medical, Japan
- ☐ Neo-Tee®, MedCare Visions® GmbH, Germany
- ☐ Neo100, monivent, Sweden
- ☐ Other: ____________

Functionality of the IVNA

Initial Neonatal Ventilation Assistant (IVNA)

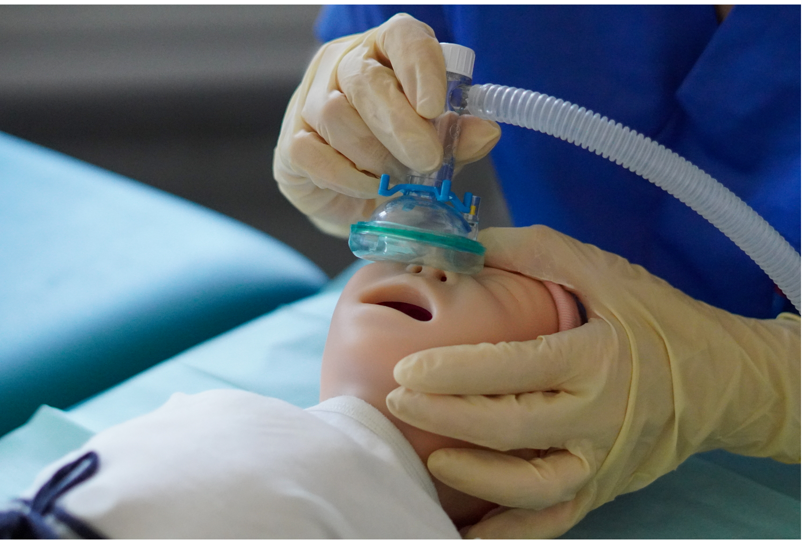

The primary objective of the research project is to create a highly advanced support system targeting a domain where human errors carry significant consequences. The central focus is on enhancing the initial care provided to newborns in the immediate postnatal period. This pertains to situations where the need for infant ventilation may arise under specific circumstances.

Current technology in neonatal ventilation relies on a tubing system with a T-piece. However, this setup lacks active mechanisms of support concerning factors such as frequency, pressure, and others. As a result, the entirety of the ventilation responsibility is borne solely by an individual.

The vision is to develop a reliable and user-intuitive assistance tool aimed at relieving medical staff from the demands of ventilation duties and mitigating the likelihood of errors. The core intention is to enable the ventilation operator to have more freedom of movement, contingent upon a successful intubation.

The rationale of the research is to find out whether and in which form support mechanisms can be integrated into the ventilation algorithms of newborns from a medical-technical point of view. Especially in the area of ventilation frequency, breath volume and ventilation pressure, protective mechanisms are to be created which are also automatically adapted to the patient. Employing the present questionnaire study, the need and the possibilities such a device should bring along to function practically should be determined.

Have you read the description of the research project? *

Please choose **only one** of the following:

- ○ Yes
- ○ No

I think *IVNA* should include the following features: *

Only answer this question if the following conditions are met:
((Description.NAOK == "Y"))

Please choose the appropriate response for each item:

	Strongly disagree 1	Disagree 2	Rather disagree 3	Neither disagree nor agree 4	Rather agree 5	Agree 6	Strongly agree 7
**automatic parameter suggestions**	○	○	○	○	○	○	○
**automated detection of infant's weight & size**	○	○	○	○	○	○	○
**pressure adaptation by device**	○	○	○	○	○	○	○
**automated pressure regulation**	○	○	○	○	○	○	○
**display of current leakage**	○	○	○	○	○	○	○
**display of current breathing volume**	○	○	○	○	○	○	○
**acoustic alarm in case of limit value exceedance**	○	○	○	○	○	○	○
**visual alarm in case of limit value exceedance**	○	○	○	○	○	○	○
**acoustic alarm in case of limit value underrun**	○	○	○	○	○	○	○
**visual alarm in case of limit value underrun**	○	○	○	○	○	○	○

	Strongly disagree<br>1	Disagree<br>2	Rather disagree<br>3	Neither disagree nor agree<br>4	Rather agree<br>5	Agree<br>6	Strongly agree<br>7
**locking of the parameter settings**	○	○	○	○	○	○	○
**maintaining the ventilation frequency at the push of a button**	○	○	○	○	○	○	○
**integrated resuscitation algorithm (similar to AED)**	○	○	○	○	○	○	○
**foot pedal for additional control**	○	○	○	○	○	○	○
**manual pushbutton for additional control**	○	○	○	○	○	○	○
**interface to work phone**	○	○	○	○	○	○	○
**interface to emergency button**	○	○	○	○	○	○	○
**interface to nurse call**	○	○	○	○	○	○	○

The following feature(s) were not mentioned, but are must-haves: *

Only answer this question if the following conditions are met:
((Description.NAOK == "Y"))

Please write your answer here:

What is your personal preference?

"1": high degree of self-control, but the device offers only limited to hardly any support (e.g. no intervention by the device if the limit value is exceeded)

"7": after configuration of your personal default settings for the automated ventilation sequence, the device takes over the execution independently (e.g. your task would be the supervision of the device)

*

Only answer this question if the following conditions are met:
((Description.NAOK == "Y"))

Choose one of the following answers
Please choose **only one** of the following:

- ◯ 1 - high degree of self-control / limited support tools
- ◯ 2
- ◯ 3
- ◯ 4
- ◯ 5
- ◯ 6
- ◯ 7 - automated ventilation sequence according to presettings

Can you describe to me your expectations regarding difficulties that might arise when using such a device (depending on the frequency of use)? *

Only answer this question if the following conditions are met:
((Description.NAOK == "Y"))

Please write your answer(s) here:

Initial Use	
Repeated Use	
Routinized Use	

What disruptions / malfunctions are strictly *not permissible* with regard to the device? *

Only answer this question if the following conditions are met:
((Description.NAOK == "Y"))

Please write your answer here:

What additional aspects should be considered in *IVNA*? *

Only answer this question if the following conditions are met:
((Description.NAOK == "Y"))

Please write your answer here:

Acceptance of the IVNA Device in Neonatal Ventilation

This is the last part of the questionnaire, please continue until the end.

Initial Neonatal Ventilation Assistant (IVNA)

Consideration of technology acceptance in combination with the integration of new functionalities plays a central role in realizing the full potential of medical devices. Successful introduction of innovative functionalities into existing medical technologies requires a thorough investigation of acceptance aspects on the part of medical professionals. By capturing the needs and requirements of users at an early stage, potential obstacles can be identified and specifically addressed.

The *IVNA* aims to provide innovative support mechanisms for ventilating premature and neonatal infants during initial care. Its objective is to maintain patient safety and uphold care at a high-quality level. Among the features it includes are the following functionalities:

- **T-piece ventilation device**
- **Integrated pressure and volume measurement:** The device is to include mechanisms for measuring ventilatory pressure and breath volume during ventilation.
- **Smart alarm management:** The device should incorporate a smart alarm system capable of responding to various scenarios.
- **Intelligent controlled ventilation function:** The ventilation function should possess intelligent control capabilities to adapt and optimize ventilation settings.
- **Automated patient recognition**

Kindly indicate your level of agreement with each statement by checking the corresponding box for each question. Some questions may be repeated intentionally to support the process of gathering information.

Please respond to the following questions based on your understanding of the *"IVNA"* and the associated research in this field. The subsequent section pertains to your perception, acceptance, and assessment of the relevance of the research work in developing such a medical device for neonatal ventilation. The practical applicability of the device is the focal point.

*

Only answer this question if the following conditions are met:
((Description.NAOK == "Y"))

Please choose the appropriate response for each item:

	Strongly disagree 1	Disagree 2	Rather disagree 3	Neither disagree nor agree 4	Rather agree 5	Agree 6	Strongly agree 7
**I would use the IVNA device for neonatal ventilation.**	○	○	○	○	○	○	○
**Using IVNA would improve the quality of neonatal ventilation.**	○	○	○	○	○	○	○
**Learning to ventilate a patient with IVNA would be easy for me.**	○	○	○	○	○	○	○

	Strongly disagree 1	Disagree 2	Rather disagree 3	Neither disagree nor agree 4	Rather agree 5	Agree 6	Strongly agree 7
**Colleagues think I should use IVNA during neonatal ventilation.**	○	○	○	○	○	○	○
**I feel confident that I can rely on the benefits during ventilation provided by the IVNA.**	○	○	○	○	○	○	○
**Using IVNA would result in my actions becoming useless during ventilation.**	○	○	○	○	○	○	○
**I would always try to use IVNA during the ventilation process.**	○	○	○	○	○	○	○
**Using IVNA would reduce likelihood of human induced errors in neonatal ventilation.**	○	○	○	○	○	○	○
**It would be easy for me to get IVNA to do what I want.**	○	○	○	○	○	○	○
**Supervisors think I should use IVNA during neonatal ventilation.**	○	○	○	○	○	○	○
**I don't think I can fully rely on the IVNA to work properly as there are too many uncertainties.**	○	○	○	○	○	○	○

	Strongly disagree 1	Disagree 2	Rather disagree 3	Neither disagree nor agree 4	Rather agree 5	Agree 6	Strongly agree 7
Having to learn how to use and adapt to IVNA would imply a significant loss of my time.	○	○	○	○	○	○	○
I intend to keep using IVNA on a regular basis.	○	○	○	○	○	○	○
Using IVNA would make it easier for me to ventilate a patient.	○	○	○	○	○	○	○
My interaction with IVNA would be clear and understandable.	○	○	○	○	○	○	○
Management of clinical institution thinks I should use IVNA during neonatal ventilation.	○	○	○	○	○	○	○
I think IVNA is a reliable tool for support of neonatal ventilation.	○	○	○	○	○	○	○
Using IVNA in my job would pose risks to neonatal patients during ventilation.	○	○	○	○	○	○	○
I would keep using the IVNA for neonatal ventilation.	○	○	○	○	○	○	○
Use of the IVNA would support decision making in patient care.	○	○	○	○	○	○	○

	Strongly disagree 1	Disagree 2	Rather disagree 3	Neither disagree nor agree 4	Rather agree 5	Agree 6	Strongly agree 7
It would be easy for me to become skillful at using IVNA.	○	○	○	○	○	○	○
Parents of patients think I should use IVNA during neonatal ventilation.	○	○	○	○	○	○	○
Using IVNA would not match my values and interests in ventilating newborns.	○	○	○	○	○	○	○
Using IVNA would enhance effective ventilation.	○	○	○	○	○	○	○
I would find it easy to adapt my ventilation workflow to IVNA.	○	○	○	○	○	○	○
Head of Department thinks I should use IVNA during neonatal ventilation.	○	○	○	○	○	○	○
Integrating the IVNA into the ventilation process would result in a loss of control.	○	○	○	○	○	○	○
I would find IVNA useful in my job.	○	○	○	○	○	○	○
I would find IVNA easy to use.	○	○	○	○	○	○	○

I would like to add the following thoughts / concerns / comments regarding the usage and usefulness of the device: *

Only answer this question if the following conditions are met:
((Description.NAOK == "Y"))

Please write your answer here:

10-30-2023 – 10:59
Submit your survey.
Thank you for completing this survey.
